# The influence of nutrition on HPV-associated inflammation: a systematic review and meta-analysis

**DOI:** 10.3389/fnut.2025.1612919

**Published:** 2025-09-19

**Authors:** Yuan Li, Lijuan Zhu

**Affiliations:** Department of Gynaecology and Obstetrics, The First People’s Hospital of Shangqiu, Shangqiu, Henan, China

**Keywords:** antioxidants, selenium, zinc, nutrition, HPV, cervical intraepithelial neoplasia, inflammation, vitamin D

## Abstract

**Background:**

Chronic infection with human papillomavirus (HPV) is a key etiologic cause of cervical intraepithelial neoplasia (CIN) and cervical cancer.

**Methodology:**

By applying MeSH terms and keywords relating to HPV, nutrition, and inflammation, sources such as PubMed, EMBASE, the Cochrane Library, and Google Scholar were examined until April 2025. Two reviewers separately selected the studies, extracted the information, and assessed the possibility of bias. Pooled estimates were computed using random-effects, with GRADE assessing confidence. 77 studies from 17 countries were included, of which the most represented were the USA (16 studies), China (12), and Iran (6). The types of studies comprised 38 case–control, 11 cross-sectional, 7 randomized controlled trials (RCTs), 7 cohort studies, and 14 nested studies. Nutrients assessed included vitamins A, C, D, E, K, B-complex (particularly B6, B12, and folate), carotenoids (*β*-carotene, lycopene, lutein), and minerals like selenium, zinc, and calcium.

**Results:**

The higher dietary intake or serum levels of micronutrients were associated with reduced persistence of HPV and decrease the risk of CIN and cervical cancer. Key findings by subgroup include: Based on 8 studies, involving 2,003 women, a protective vitamin E (particularly *α*-tocopherol) effect against HPV and cervical neoplasia (SMD = 0.46 [0.36, 0.57]; *p* < 0.0001; I^2^ = 21.3%; GRADE: Moderate) was noted. Across 5 studies, including 727 women, both oral and vaginal vitamin D supplementation reduced CIN2/3 lesions and improved inflammatory markers (SMD = 0.81 [0.69, 0.93]; *p* < 0.00001; I^2^ = 0%; GRADE: High). From 6 studies (1,246 individuals) the consistent inverse associations between vitamin A intake/status and risk of cervical cancer (SMD = 0.77 [0.68, 0.87]; *p* < 0.0001; I^2^ = 16.6%; GRADE: Moderate) was observed. Across 4 studies (1,130 women), folate and Vitamin B12 showed protective role in reducing HPV persistence and CIN progression, with favorable effects on DNA methylation and viral clearance (SMD = 0.80 [0.65, 0.95]; *p* < 0.00001; I^2^ = 0%; GRADE: High). Selenium supplementation, notably in Iranian trials (GRADE: Moderate) improved oxidative and immune profiles and was associated with CIN2 regression. Zinc and calcium were associated with immune enhancement and viral suppression (GRADE: Low to moderate).

## 1 Introduction

One of the biggest contributors of cancer-related death for women globally, especially in nations with low or moderate incomes, is cervical cancer (CC). Cervical cancer and cervical intraepithelial neoplasia (CIN) have been reported to be caused by persistent contact with the high-risk human papillomavirus (hrHPV) (1). Despite cytological testing and preventative HPV vaccination have greatly decreased the prevalence of the illness in wealthy areas, their worldwide impact is still below ideal owing to facilities, easy access, and financial constraints. In order to reduce HPV prevalence and the ensuing cancer development, there is growing interest in complimentary and controllable variables, such as a nutritious diet. Nutrition and diet are essential for preserving general health and averting a number of illnesses. The immune system, which is crucial for fending off ailments, including HPV infections, and stopping the development and spread of cancer cells, can be strengthened by eating a nutritious diet. Thus, maintaining a balanced diet is crucial to halting the development of cervical cancer (CC) from a chronic high-risk human papillomavirus (hrHPV) infection (2). A number of protective factors have been identified as potentially reducing the risk of cervical cancer folate, vitamin A, calcium, vitamin D, vitamins C and B12, as well as *β*-carotene, and trace minerals including zinc and selenium. Folate, for instance, may help reduce the incidence of cervical cancer (CC). Folate, for example, may slow the evolution of cervical lesions by controlling DNA methylation and gene expression linked to tumor suppression. Likewise, vitamin B12 and folate regulate DNA synthesis and repair processes in concert (1, 3, 4). A greater probability of cervical intraepithelial neoplasia (CIN) in women infected with hrHPV has been linked to vitamin deficiencies. Vitamins C, A, and E that are antioxidants also help to prevent oxidative harm to DNA and boost immunological reactions that are essential for the removal of viruses (5). The antioxidant activity and immunomodulatory properties of trace minerals including zinc and selenium have been shown in recent research to protect against carcinogenesis, especially in the pathogenesis of cervical neoplasia. While zinc supplementation has been attributed with better HPV clearance and lesion regression, selenium has shown positive impacts in lessening oxidative damage and encouraging CIN regression (6, 7). According to this data, these micronutrients could influence inflammatory markers and help afflicted people achieve more favorable clinical results. Although the relationship between diet and HPV is gaining focus, the data that currently exists is still inconsistent, with results differing depending on the demographic, methodology of research, nutritional evaluation techniques, and results criteria. Previous research has often lacked the ability to identify modest correlations or evaluate inflammatory biomarkers as intermediary targets in the HPV infection-to-neoplasia process. A thorough synthesis of statistical data connecting dietary intakes to HPV-associated inflammatory conditions, a key molecular contributing factor to cervical carcinogenesis, has also not been done previously. This gap is filled by the current systematic examination and meta-analysis, which rigorously assesses how food and particular micronutrients affect the inflammatory reactions linked to hrHPV illness. In order to increase quantitative accuracy, elucidate the cause and effect of relationships, and provide a solid, information-based knowledge of how diet may alter HPV-related immunological processes, our effort will integrate findings from observational investigations and clinical trials. Thus, our research offers contemporary and therapeutically applicable data that might guide preventative measures in situations when traditional therapies are still unattainable.

## 2 Materials and methods

The PRISMA 2020 guidelines were applied in the execution of this systematic review and meta-analysis.

### 2.1 Search strategy

The Cochrane Library, Google Scholar, PubMed (Medline), and EMBASE databases were searched from the inception dates to April 2025. Considering the large amount of gray and non-peer-reviewed publications on Google Scholar, two independent reviewers meticulously filtered the first 200 results, according to the abstract and title’s relevancy. Full texts of research papers that might be appropriate were obtained. The following were the primary search terms used for article texts, abstracts, or MeSH headings: HPV, human papillomavirus, nutrition, dietary intake, micronutrient intake, vitamin supplementation, inflammation, anti-inflammatory diet, cytokine expression, immune response, antioxidants, oxidative stress, randomized controlled trial, clinical trial, and interventional study. The search criteria were modified by integrating these phrases utilizing the Boolean logic operator AND. While searching the database, a language limitation was implemented; only English-language publications were taken into consideration for inclusion (Table 1).

**Table 1:** Comprehensive electronic search methods for systematic review.

Database	Search strategy	Last search
PubMed (MEDLINE)	“human papillomavirus”[Mesh] OR HPV OR “human papillomavirus infection” AND “nutrition”[Mesh] OR “dietary intake” OR AND “inflammation”[Mesh] OR “anti-inflammatory diet” OR “immune response” OR “cytokine expression” OR “oxidative stress” OR “antioxidants” AND “clinical trial “OR “randomized controlled trial”	April, 2025
EMBASE	‘human papillomavirus’/exp. OR hpv) AND ‘dietary intake’/exp. OR ‘nutrition’/exp. OR ‘vitamin supplementation’/exp. OR ‘micronutrient intake’/exp. AND ‘inflammation’/exp. OR ‘anti-inflammatory diet’/exp. OR ‘immune response’/exp. OR ‘cytokine expression’/exp. OR ‘oxidative stress’/exp. OR “antioxidants’/exp. AND (‘clinical trial’/exp. OR ‘randomized controlled trial’/exp)	April, 2025
Cochrane Library (CENTRAL)	“HPV” OR “human papillomavirus” AND “dietary intake” OR “nutrition” OR “vitamin supplementation “OR “micronutrient intake” AND “immune response” OR “inflammation” OR “cytokine expression” OR “antioxidants” OR “oxidative stress”	April, 2025
Google Scholar	“HPV” OR “human papillomavirus” AND “dietary intake” OR “nutrition” OR “vitamin supplementation” OR “micronutrient intake” AND “inflammation” OR “cytokine expression” OR “immune response” OR “antioxidants” OR “oxidative stress” AND “clinical trial” OR “randomized controlled trial”	April, 2025

### 2.2 Inclusion criteria

Studies were included based on predefined inclusion and exclusion criteria structured using population, Intervention, Control, Outcome, and Study (PICOS) (Table 2). Studies involving patients diagnosed with HPV infection that investigated the influence of dietary intake or nutrient supplementation on inflammatory markers or HPV associated outcomes were included. Studies were excluded if they were *in vitro*, focused on vaccine response without nutritional or dietary data or involved participants with co-existing chronic conditions (Cancers, autoimmune disorders etc).

**Table 2:** Summary of PICOS criteria.

Element	Scope
Population	Patients with documented HPV Infection (particularly females with cervical HPV), With or without CIN
Intervention	Dietary intake or micronutrient supplementation
Comparator	Placebo (RCTs), no supplement/usual care
Outcomes	HPV perseverance or elimination and virus load; prevalence, development, or decline of CIN; alterations in indicators of oxidative damage or inflammation
Study design	RCTs, nested, case–control cohort, and cross-sectional designs

### 2.3 Outcomes and measures

The well-known natural trajectory of HPV can be divided into three categories of outcomes: mechanistic (alterations in oxidative damage or pro-inflammatory markers), precancerous (development, growth, or regression of CIN), and virologic (acquisition, perseverance, or removal of infection). We chose this paradigm since studies usually provide results at all three phases, oxidative damage and inflammatory signaling are crucial stages in the onset of the illness, and persistent high-risk HPV is the main contributory factor of CIN and cervical cancer.

### 2.4 Data extraction

Two researchers separately assessed every piece of possibly pertinent literature, and they all came to a consensus. The first author, publication year, study design (randomized controlled trials, prospective or retrospective studies, and case control studies), sample size, demographic information (age, sex), enrollment period, HPV infection status, dietary assessment type and method, specific nutrients, patterns evaluated or dietary components, inflammatory markers, and type and site of HPV infection were all extracted with respect to each included study.

### 2.5 Assessment bias

By employing the Newcastle-Ottawa Scale (NOS) for observational research and the Cochrane Risk of Bias Tool 2.0 for randomized controlled trials, the risk of bias was determined. Along with domain-driven evaluations (such as reporting, detection, and performance bias), we also categorized investigations into low, moderate, and high risk of bias according to the three fundamental areas that are thought to be essential to internal validity: (1) concealing allocations (selection bias); (2) blinding the subjects and examiners (performance/detection bias); and (3) attrition bias (insufficient end result information). These cutoff points were modified from Cochrane RoB 2.0 recommendations to make subgroup examinations and sensitivity evaluations easier. Any disagreements between the two researchers about the choice of study or the extraction of data were either settled by consensus or forwarded to a third researcher.

### 2.6 Certainty of evidence

We employed the GRADE (Grading of Recommendations Assessment, Development, and Evaluation) framework covering five domains—risk of bias, inconsistency, indirectness, imprecision, and publication bias—to determine the total validity of the evidence.

### 2.7 Statistical analysis

For the purpose of statistical analysis, the Cochrane Software Review Manager (version 5.3, The Cochrane Collaboration, Copenhagen, Denmark) was deployed. For continuous variables, pooled effect sizes were determined as standardized mean differences (SMDs); for binary outcomes, they were computed as odds ratios (ORs), each with an interval of confidence of 95%. The random-effects model (DerSimonian and Laird approach) was implemented to consider inter-study variation into consideration. The I^2^ statistic has been employed to measure heterogeneity; numbers greater than 50% indicated substantial variation. We employed meta-regression to look into possible causes of variance when the heterogeneity in the results was substantial (I^2^ above 70%). Predetermined subgroup analyses were performed with regard to the study design, HPV infection site, and nature of dietary intake. To determine the accuracy of the findings, sensitivity analyses was carried out through removing papers with outlier effect sizes or a high risk of bias. Graphical investigation of funnel plots was used to figure out publication bias, and when a minimum of 10 studies could be accessed, Egger’s regression asymmetry test was employed to perform statistical testing. A two-tailed *p*-value of below 0.05 was considered statistically significant.

### 2.8 Sensitivity and influence analyses

Sensitivity and influence evaluation was carried out to make sure the outcomes were valid. To determine its impact on the pooled estimates, a leave-one-out sensitivity analysis was applied by successively eliminating every investigation. Meta-analyses with and without studies classified as high risk were compared in order to perform a high-risk bias exclusion analysis. Furthermore, subgroup analyses were also carried out according to the form of nutrients (vitamin D vs. vitamin E vs. others), the location of HPV infection (cervical vs. oropharyngeal), and the study method (RCT vs. observational).

## 3 Results

**Figure 1:**
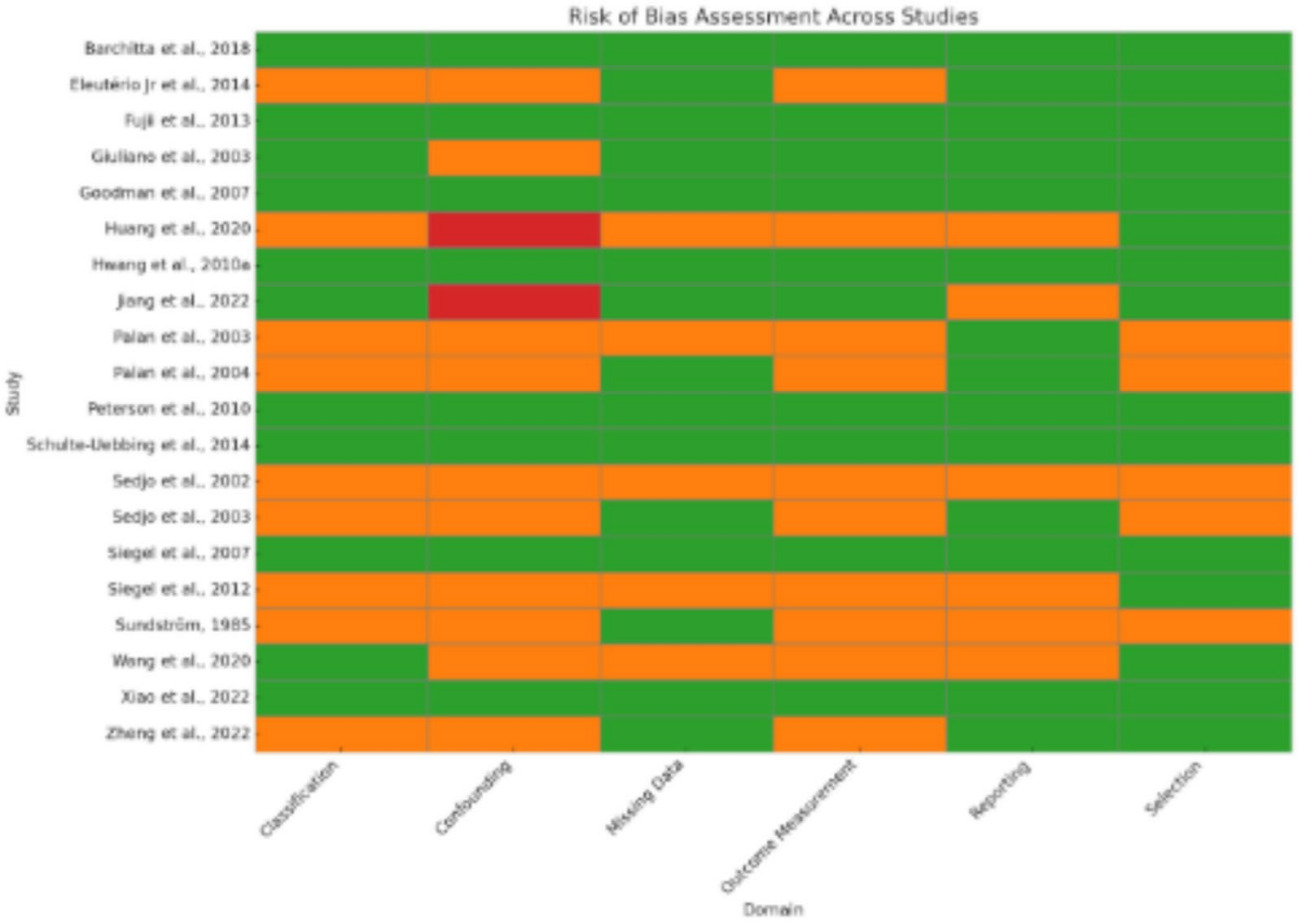
Risk bias for observational studies.

77 studies were included in this meta-analysis. These studies were conducted in 17 countries, most of which (16 studies) were represented from the USA, followed by China (12 studies) and Iran (6 studies). Other countries contributing to the pool were Brazil, India, Japan, Korea, Italy, and various European and South American countries, indicating an interest around the world in learning about the potential role of nutrition in the prevention and treatment of HPV-related diseases. The studies used a range of methodological designs. The most common (38 studies) were case–control studies, which mostly compared nutrient consumption or serum level of nutrients among individuals with or without HPV infection or cervical intraepithelial neoplasia (CIN). Cross-sectional studies (11 studies) explored associations at a single time point between micronutrient status and HPV-related outcomes (Table 1; Figure 1).

**Figure 2:**
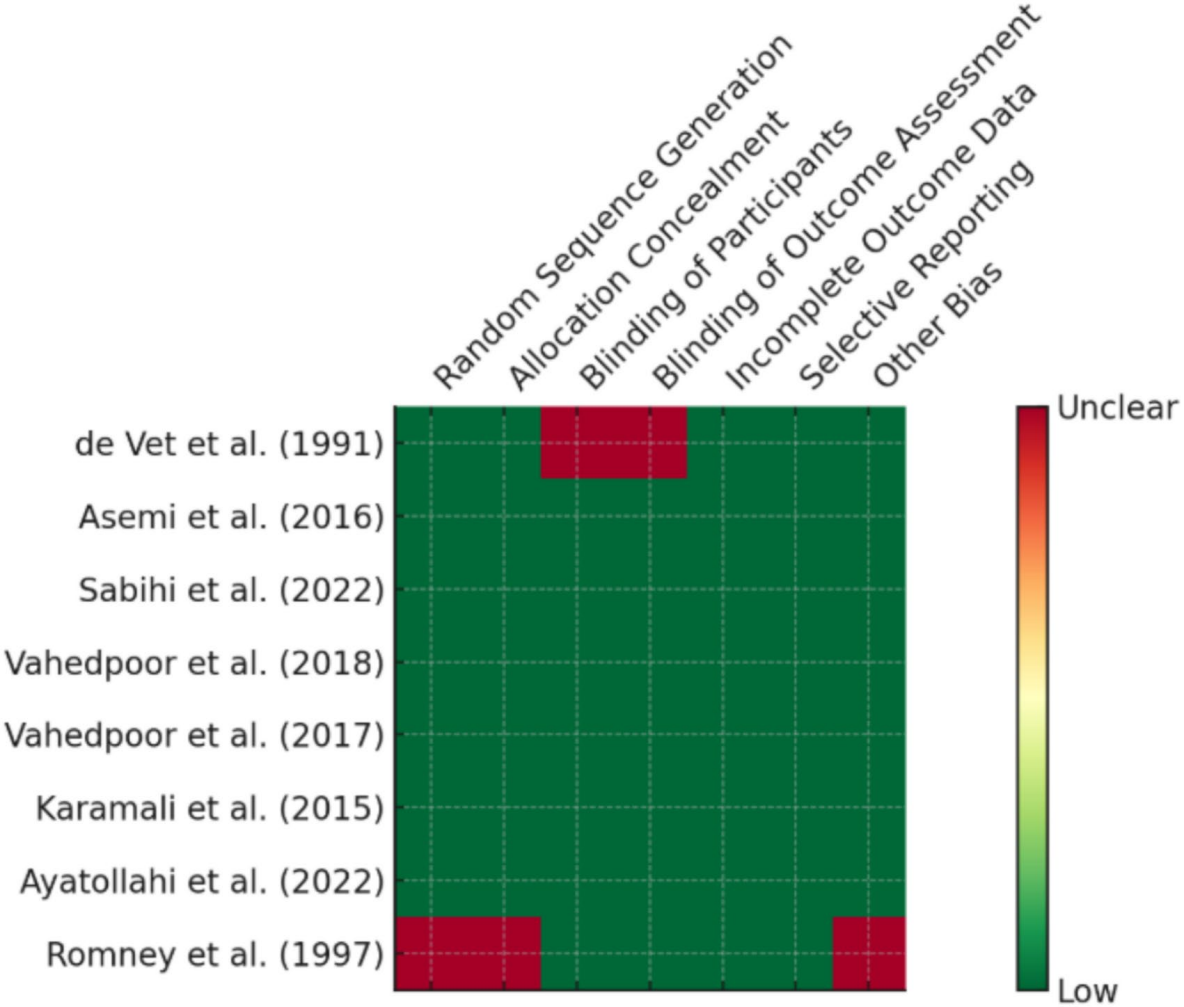
Risk of bias assessment for RCTs.

**Figure 3:**
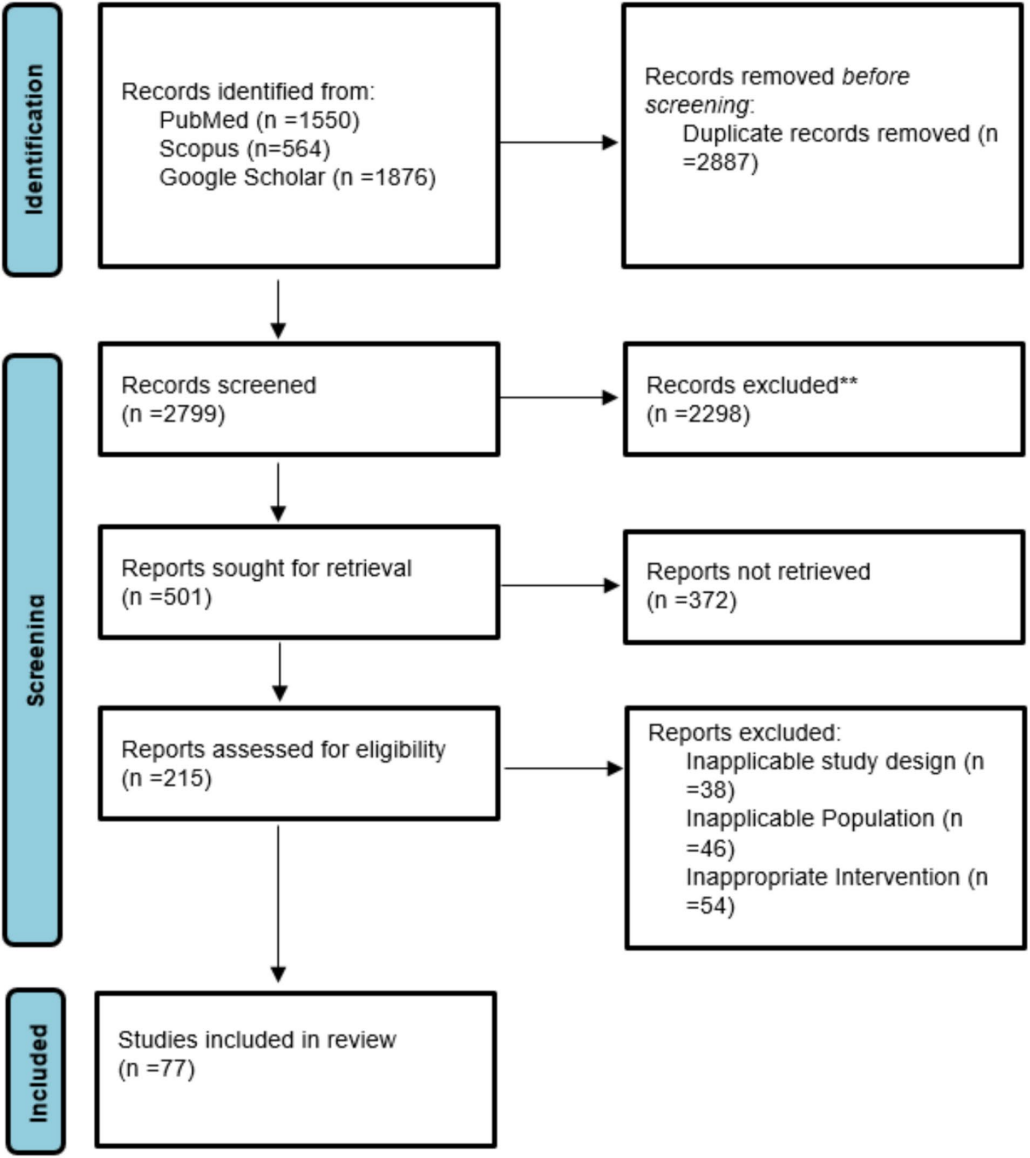
PRISMA flow chart of data selection.

Randomized controlled trials (7 studies), which were mainly from Iran and the USA, provided high-level evidence of the efficacy of some supplements such as vitamin D, selenium, and folate in promoting CIN regression and modulation of inflammatory biomarkers (Figure 2). Additionally, 7 cohort or prospective studies followed the population longitudinally to study the impact of nutritional status on persistence of HPV and development of CIN. The remaining 14 studies presented a combination of ecological studies, and nested case–control designs. Figure 3 shows the PRISMA flow diagram and details the extensive selection procedure of the incorporated investigation (Tables 3–11).

**Table 3:** Vitamin E.

Author, year	Country	Study design	Sample size	Supplement	Intervention	Vitamin/antioxidant assessed	Outcome related to the influence of nutrition on HPV-associated inflammation
Guo et al. (20)	China	Case–control study	458/742	Vitamin E	Serum levels, dietary intake	Vitamins A, C, E	Higher antioxidant intake linked to reduced cervical cancer risk via potential anti-inflammatory effects.
Zhang et al. (21)	China	Case control study	158/200	Vitamin E	Serum micronutrients	Carotenoids, Retinol, Tocopherols	Lower serum levels associated with higher cancer risk, supporting role in HPV-related inflammation modulation.
Tomita et al. (22)	Brazil	Case control study	453/605	Vitamin E	Serum and dietary micronutrients	α-Tocopherol, *β*-carotene, Lycopene	Lower antioxidant levels associated with CIN risk; antioxidants may suppress HPV-triggered inflammation.
Ghosh et al. (14)	USA	Case control study	239/979	Vitamin E	Dietary intake	Vitamins A, C, E	Dietary antioxidants inversely associated with cervical cancer risk, possibly mitigating HPV-driven inflammation.
Siegel et al. (15)	USA	Cohort study	229/405	Vitamin E	Serum concentrations	Carotenoids, Tocopherols	High serum antioxidant levels reduced HPV persistence, likely due to modulation of inflammatory response.
Palan et al. (23)	USA	Cross sectional study	21/51	Vitamin E	Plasma antioxidant levels	*α*-Tocopherol, *α*-Tocopheryl Quinone	Decreased α-tocopherol and increased oxidized metabolites linked to cervical neoplasia and inflammation.
Palan et al. (24)	USA	Cross sectional study	48/162	Vitamin E	Plasma levels of antioxidants	Coenzyme Q10, α- and *γ*-Tocopherols	Lower plasma levels observed in cervical neoplasia cases; potential protective antioxidant role against HPV-related inflammation.
Goodman et al. (25)	USA	Case control study	147/191	Vitamin E	Serum micronutrient concentrations	*β*-Carotene, Lutein/Zeaxanthin, Lycopene, Tocopherols	Higher serum antioxidant levels associated with faster clearance of oncogenic HPV infections.

**Table 4:** Vitamin K.

Author, year	Country	Study design	Sample size	Supplement	Intervention	Nutrient assessed	Outcome related to the influence of nutrition on HPV-associated inflammation
Jiang et al. (18)	China	Cross sectional study	13,447	Vitamin K	Dietary intake	Vitamin K	Lower dietary vitamin K intake significantly associated with higher HPV infection rates.
Wang et al. (19)	China	Cohort study	218	Vitamin K	Dietary nutrient intake	Vitamins A, C, E, B12, Folate	Lower intake of key vitamins (esp. B12, folate, A) associated with higher CIN risk; nutrients may reduce HPV-related inflammation.

**Table 5:** Vitamin D.

Author, year	Country	Study design	Sample size	Supplement	Intervention	Vitamin/antioxidant assessed	Outcome related to the influence of nutrition on HPV-associated inflammation
Vaheedpoor et al. (10)	Iran	RCT	58	Vitamin D	Vitamin D3 (50,000 IU biweekly)	Vitamin D	Vitamin D supplementation led to improved metabolic markers and reduced CIN 2/3 recurrence, indicating anti-inflammatory benefit.
Vaheedpoor et al. (9)	Iran	RCT	58	Vitamin D	Vitamin D3 (50,000 IU biweekly)	Vitamin D	Significant CIN regression and metabolic improvement observed; supports anti-inflammatory role in HPV-infected tissue.
Özgü et al. (26)	Turkey	Case control study	23/62	Vitamin D	Serum 25-OH Vitamin D levels	Vitamin D	Vitamin D deficiency associated with persistent HPV infection in women with cervical lesions.
Schulte-Uebbin et al. (27)	Germany	Cross sectional study	100/200	Vitamin D	Vaginal vitamin D suppositories (12,500 IU daily)	Vitamin D	High-dose vaginal vitamin D resolved HPV and CIN lesions in most participants, suggesting potent local anti-inflammatory effect.
Hosono et al. (28)	Japan	Case control study	405/2,025	Vitamin D	Dietary intake assessment	Vitamin D, Calcium	Higher vitamin D intake inversely associated with cervical neoplasia; potentially protective via immune and inflammatory modulation.

**Table 6:** Vitamin C.

Author, year	Country	Study design	Sample size	Supplement	Intervention	Vitamin/antioxidant assessed	Outcome related to the influence of nutrition on HPV-associated inflammation
Zheng et al. (5)	China	Cross sectional study	217	Vitamin C	Serum vitamin C levels	Vitamin C	Lower serum vitamin C levels associated with increased HPV infection prevalence; implicates antioxidant role in infection control.
Barchitta et al. (2)	Italy	Cross sectional study	251	Vitamin C	Dietary pattern analysis	Multiple antioxidants via diet	Healthy dietary patterns rich in antioxidants inversely associated with high-risk HPV infection and cervical cancer risk.
Naidu et al. (29)	India	Case control study	30/120	Vitamin C	Oxidative stress markers and antioxidants	Vitamins C, E, Glutathione	Cervical cancer patients showed elevated oxidative stress and decreased antioxidants, indicating inflammation role.
Giuliano et al. (30)	USA	Nested case study	185/248	Vitamin C	Dietary intake and serum micronutrients	Vitamins A, C, E, Carotenoids	Persistent HPV infection associated with lower antioxidant intake; antioxidants may aid immune response and inflammation control.
Shannon et al. (31)	Thailand	Case control study	134/348	Vitamin C	Dietary intake	Vitamins A, C, Carotenoids	Dietary deficiencies linked to increased cervical carcinoma risk; antioxidants may reduce HPV-related inflammation and carcinogenesis.
Herrero et al. (32)	Costa Rica	Case control study	141/748	Vitamin C	Dietary intake	Vitamins A, C, Riboflavin, Folate	Lower nutrient intake related to increased invasive cervical cancer risk, possibly via immune and inflammatory pathways.
Ziegler et al. (33)	USA	Case control study	229/502	Vitamin C	Dietary intake	Vitamins A, C, Carotenoids	Inverse association of vitamin A and carotenoids with in situ cervical cancer, implicating protective inflammatory modulation.
Brock et al. (34)	Australia	Case control study	117/196	Vitamin C	Dietary and plasma nutrients	Vitamins A, C, E	Lower plasma vitamin concentrations associated with higher in situ cervical cancer risk; antioxidants may limit HPV-related inflammation.

**Table 7:** Vitamin A.

Author, year	Country	Study design	Sample size	Supplement	Intervention	Vitamin/antioxidant assessed	Outcome related to the influence of nutrition on HPV-associated inflammation
Huang et al. (35)	China	Cross sectional study	13,412	Vitamin A	Dietary vitamin A intake	Vitamin A	Low vitamin A intake correlated with increased HPV infection prevalence; vitamin A may influence inflammatory response.
Eleutério et al. (36)	Brazil	Cross sectional study	62	Vitamin A	Serum vitamin A levels	Vitamin A	Low serum vitamin A levels significantly associated with high-grade squamous intraepithelial lesions; implicates immune/inflammation pathways.
Kim et al. (16)	Korea	Case control study	144/288	Vitamin A	Zinc-citrate compound CIZAR® topical treatment	Zinc	Zinc treatment reduced HPV viral load and cervical lesion severity, indicating anti-inflammatory and antiviral potential.
Yeo et al. (37)	USA	Case control study	190/326	Vitamin A	Serum micronutrient measurement	Vitamins A, C, E, Zinc, Selenium	Lower serum micronutrients associated with cervical dysplasia; zinc and antioxidants may modulate inflammation in HPV infection.

**Table 8:** Selenium.

Author, year	Country	Study design	Sample size	Supplement	Intervention	Vitamin/antioxidant assessed	Outcome related to the influence of nutrition on HPV-associated inflammation
Karamali et al. (38)	Iran	RCT	28/58	Selenium	Selenium supplementation 200 μg/day	Selenium	Selenium supplementation promoted regression of CIN and improved metabolic profiles; anti-inflammatory effects suggested.
Cunzhi et al. (39)	China	Case control study	50/70	Selenium	Serum and tissue trace elements	Zinc, Copper, Selenium	Altered trace element levels and Cu/Zn ratio in cervical cancer patients suggest oxidative stress and inflammatory involvement.
Xiao et al. (40)	China	Cross sectional study	4,628	Selenium	Dietary intake assessment	Zinc, Copper, Selenium	Higher dietary zinc and selenium intake inversely associated with high-risk HPV infection; nutrients may reduce inflammation.
Obhielo et al. (7)	Nigeria	Case control study	45	Selenium	Serum selenium measurement	Selenium	Lower serum selenium levels associated with higher CIN risk; selenium may have anti-inflammatory role in cervical neoplasia.
Shimizu et al. (17)	Japan	Case control study	137	Vitamin A	Serum retinol measurement	Vitamin A retinol	Women with cervical dysplasia showed decreased serum retinol, indicating antioxidant deficiency may influence HPV inflammation.
Sundström et al. (41)	Finland	Cross sectional study	25/32	Selenium	Serum selenium levels monitored annually	Selenium	Annual selenium variation noted; low selenium associated with gynecological cancers including cervical lesions, linked to oxidative stress.

**Table 9:** Calcium, iron and zinc.

Author, year	Country	Study design	Sample size	Supplement	Intervention	Vitamin/antioxidant assessed	Outcome related to the influence of nutrition on HPV-associated inflammation
Hwang et al. (42)	Korea	Cross sectional study	256/328	Calcium	Dietary supplement use questionnaire	Multivitamins and antioxidants	Supplement use linked to decreased risk of CIN, suggesting a protective anti-inflammatory effect.
Siegel et al. (43)	USA	Cohort study	327	Iron	Serum iron biomarker measurement	Iron status biomarkers	Higher iron storage biomarkers associated with altered HPV clearance, implying iron-related oxidative stress in HPV persistence.
He et al. (44)	China	Cross sectional study	13,475	Calcium	Dietary calcium intake questionnaire	Calcium	Higher calcium intake associated with reduced HPV infection prevalence, suggesting anti-inflammatory effects of calcium.
Ayatollahi et al. (6)	Iran	RCT	40/40	Zinc	Oral zinc sulfate supplementation	Zinc	Zinc supplementation significantly increased HPV clearance rate, indicating zinc’s immunomodulatory and anti-inflammatory role.
Chen et al. (13)	Taiwan	Case control study	50/90	Zinc	Serum copper and zinc measurement	Copper, Zinc	Altered serum copper and zinc levels observed in cervical cancer patients; imbalance may contribute to oxidative stress and HPV inflammation.
Sengupta et al. (45)	India	Cross sectional study	28/28	Calcium	Serum calcium measurement	Calcium	Serum calcium levels correlated with cervical cancer status; potential link to inflammatory pathways in cervical carcinogenesis.
Grail and Norval (46)	Scotland	Case control study	21/110	Zinc	Serum copper and zinc measurement	Copper, Zinc	Differences in serum copper and zinc between patients with cervical abnormalities and controls; possible role in modulating HPV-associated inflammation.
Liu et al. (47)	USA	Case–control study	523	Calcium	Dietary questionnaires	Various nutritional factors including folate, vitamins A and C	Nutritional deficiencies linked to increased cervical dysplasia risk, with folate deficiency showing strong association with HPV-related inflammation.

**Table 10:** Vitamin B12.

Author, year	Country	Study design	Sample size	Supplement	Intervention	Vitamin/antioxidant assessed	Outcome related to the influence of nutrition on HPV-associated inflammation
Li et al. (48)	China	Case control study	80/174	B12	Folate status measurement	Folate	Folate deficiency correlated with aberrant DNA methylation in FHIT gene, promoting cervical pathogenesis and HPV-associated inflammation.
Sabihi et al. (49)	Iran	RCT	60	B12	Folate supplementation	Folate	Folate supplementation reduced recurrence of cervical intraepithelial neoplasia and improved metabolic status, suggesting anti-inflammatory effects.
Zhao et al. (4)	China	Cross sectional study	877/20,000	B12	Folate status measurement	Folate	Low folate status associated with higher risk of cervical intraepithelial neoplasia, implicating folate in modulating HPV-induced inflammation.
Piyathilake et al. (50)	India	Cross sectional study	16/315	B12	Dietary folate and vitamin B12 assessment	Folate, Vitamin B12	Folate and vitamin B12 inversely associated with HPV 16 methylation and higher-grade CIN risk, suggesting their protective role in HPV-related inflammation.
Piyathilake et al. (3)	India	Cross sectional study	724	B12	Plasma folate and vitamin B12 measurement	Folate, Vitamin B12	High plasma folate with sufficient B12 linked to lower risk of cervical intraepithelial neoplasia, suggesting protective effects on HPV inflammation.
Bai et al. (51)	China	Cross sectional study	109/202	B12	Folate deficiency assessment, HPV 16 infection status	Folate	Folate deficiency and FHIT gene hypermethylation synergistically promoted HPV 16-related cervical cancerization via inflammation pathways.
Ragasudha et al. (52)	India	Case control study	136/186	B12	Folate and vitamin B12 nutritional status	Folate, Vitamin B12	Synergistic interaction of folate and B12 influenced cervical cancer progression through epigenetic and inflammatory mechanisms.
Abike et al. (11)	Turkey	Case control study	122	B12	Measurement of neopterin, folate, homocysteine levels	Folate, Homocysteine	Persistent HPV infection associated with altered folate and neopterin levels, indicating links to immune activation and inflammation.
Asemi et al. (8)	Iran	RCT	58	B12	Long-term folate supplementation	Folate	Folate supplementation improved metabolic profiles and promoted regression of cervical intraepithelial neoplasia, reducing HPV-associated inflammation.
Wang et al. (53)	China	Case control study	111/111	B12	Folate measurement	Folate	Lower folate levels associated with increased cervical cancer risk, indicating folate’s role in modulating HPV-related inflammation.
Hernandez et al. (54)	USA	Case control study	214/217	B12	Dietary intake assessment	Folate, Riboflavin, Thiamin, Vitamin B12	Diets rich in folate and B vitamins were protective against premalignant cervical lesions, reducing HPV-induced inflammation.

**Table 11:** Fruits and vegetables.

Author, year	Country	Study design	Sample size	Supplement	Intervention	Vitamin/antioxidant assessed	Outcome related to the influence of nutrition on HPV-associated inflammation
Fuji et al. (55)	Japan	Prospective study	391	Carotenoids	Carotenoid blood levels	Carotenoids	Higher carotenoid levels associated with better CIN outcomes, suggesting antioxidant role in HPV-associated inflammation control.
Peterson et al. (56)	USA	Prospective study	120/141	Carotenoids	Combined carotenoid antioxidants	Carotenoids	Combined carotenoid antioxidants linked to reduced risk of persistent HPV infection through anti-inflammatory mechanisms.
Tomita et al. (57)	Brazil	Case control study	231/453	Carotenoids	Dietary intake of dark-green and deep-yellow vegetables/fruits	Carotenoids, Vitamins	Vegetable and fruit intake inversely associated with CIN, with effects modified by smoking status, likely via inflammatory pathways.
Sedjo et al. (58)	USA	Prospective study	84	Carotenoids	Plasma micronutrient measurement	Various micronutrients	Higher plasma micronutrient levels associated with increased clearance of oncogenic HPV, indicating anti-inflammatory roles.
Sedjo et al. (59)		Prospective study	1,042	Carotenoids	Vitamin A and carotenoid levels	Vitamin A, Carotenoids	Lower vitamin A and carotenoid levels linked to increased risk of persistent oncogenic HPV infection.
Schiff et al. (60)	USA	Case control study	81/160	Carotenoids	Serum carotenoid levels	Carotenoids	Higher serum carotenoids associated with reduced risk of cervical intraepithelial neoplasia via anti-inflammatory effects.
Nagata et al. (1)	Japan	Case control study	152/152	Carotenoids	Serum carotenoids and vitamin levels	Carotenoids, Vitamins	Lower serum carotenoids and vitamins associated with increased risk of cervical dysplasia.
Palan et al. (61)	USA	Case control study	95/140	Carotenoids	Plasma levels of carotenoids, retinol, tocopherols	Beta-carotene, Lycopene, Canthaxanthin, Retinol, α−/*τ*-Tocopherol	Reduced plasma antioxidant levels linked to cervical intraepithelial neoplasia and cancer, implicating oxidative stress and inflammation.
Kanetsky et al. (62)	USA	Case control study	60/113	Carotenoids	Dietary intake and blood lycopene levels	Lycopene	Higher lycopene intake and blood levels linked with reduced cervical dysplasia risk, suggesting anti-inflammatory effects.
Romney et al. (63)	USA	RCT	69	Carotenoids	β-Carotene supplementation and other factors	β-Carotene	β-Carotene supplementation associated with regression of cervical dysplasia and reduced HPV infection persistence.
De Vet et al. (64)	Netherlands	Case control study	257	Carotenoids	β-Carotene supplementation	β-Carotene	β-Carotene supplementation showed effects on regression and progression of cervical dysplasia.
dE Vet et al. (65)	Netherlands	RCT	137/141	Carotenoids	Dietary intake assessment	*β*-Carotene and dietary factors	Lower dietary β-carotene linked with higher cervical dysplasia risk.
Gonzalez et al. (66)	Case–control dysplasia			Tomatoes and fruits	Dietary intake self-reported FFQ	Vitamins C, E, retinol, β-carotene, folate	Higher intake of vitamin C, retinol, and folate inversely associated with cervical cancer risk; suggests protective role against HPV-related inflammation.
Cuzik et al. (67)	London	case–control Cervical cancer	121/241	Fruits and leafy vegetables	Observational dietary/lifestyle assessment	Vitamin A, C, folate	Lower intake of folate and vitamin C associated with increased cervical cancer risk; indicates nutritional deficiencies may promote HPV persistence and inflammation.
Harris et al. (68)	UK	Case control study	226/226	Carotenoids	Observational blood and dietary vitamin A levels	Vitamin A serum retinol and dietary intake	No clear association between dietary vitamin A and cervical cancer; slight inverse trend in serum retinol but not statistically significant—limited support for anti-inflammatory role.
Van Eenwyk et al. (69)	USA	Case control study	102/102	Carotenoids	Observational dietary intake and serum analysis	β-carotene, lutein, lycopene, other carotenoids	Lower serum carotenoid levels especially β-carotene and lutein associated with higher risk of CIN; suggests carotenoids may reduce HPV-induced oxidative stress and inflammation.
dE Vet et al. (65)	USA	Case control study	50/100	Carotenoids	Dietary intake assessment	β-Carotene and dietary factors	Lower β-Carotene intake linked with higher cervical dysplasia risk.
Potischman et al. (70)	USA	Case control study	67/387	Carotenoids	Serologic nutrient indicators	Various nutrients	Lower serum nutrient levels linked to invasive cervical cancer risk.
Verreault et al. (71)	USA	Cervical Carcinoma case–control	189/227	Vegetables, fruits, and dark green and yellow	None Observational	Multiple nutrients including vitamins and antioxidants	Diet quality associated with invasive cervical cancer risk; certain nutrients may modulate HPV-related inflammation and carcinogenesis.
Giuliano et al. (72)	China	Cervical cancer case–control	101/146	Veggies	None Literature review	Multiple nutrients including antioxidants, vitamins A, C, E	Nutrients play a significant role in preventing cervical dysplasia and cancer, potentially through modulation of HPV infection and inflammation pathways.
Rajkumar et al. (73)	India	Cervical cancer case–control	205/213	Fruits and vegetables, green veggies, fresh tomatoes, carrots, and cruciferous	None Observational	No specific vitamin; focused on dietary habits and paan chewing	Paan chewing and poor dietary habits associated with increased cervical carcinoma risk, potentially through HPV-related inflammation and carcinogenesis pathways.
Atalah et al. (12)	Chile	case–control	170/340	Leafy veggies and carrots	None Observational	No specific vitamin or antioxidant	Diet, smoking, and reproductive history were identified as risk factors for cervical cancer, suggesting nutrition’s influence on HPV-associated inflammatory pathways.
Vecchia et al. (74)	Italy	case–control Cervical cancer	392/392	Carrots,	None Observational	Vitamin A	Low dietary vitamin A intake associated with increased risk of invasive cervical cancer, implicating vitamin A’s role in modulating HPV-associated inflammation and carcinogenesis.
Larrinaga et al. (75)	Uruguay	case–control Cervical cancer	53/208	Veggies	None Observational	Not specified	Parity, sexual factors, and dietary habits associated with cervical cancer risk, indicating nutrition’s role in HPV-related inflammation and cancer development.
Hwang et al. (76)	Korea, Nested	case–control CIN 2/3 only HPV+	162/166	Fruits and vegetables	None Observational	Antioxidants and micronutrients in fruits and vegetables	Higher fruit and vegetable intake associated with lower HPV viral load in high-risk HPV-positive women with cervical intraepithelial neoplasia, suggesting reduced HPV-related inflammation risk.
Hirose et al. (77)	Japan	case–control Cervical cancer	416/20,985	Carrots, green veggies, and fruits	None Observational	Vitamins A, C, E, and smoking status	Smoking and low intake of vitamins A, C, E were associated with increased cervical cancer risk, suggesting their role in modulating HPV-associated inflammation and carcinogenesis, with age-specific effects.

### 3.1 Risk of bias across studies

**Figure 4:**
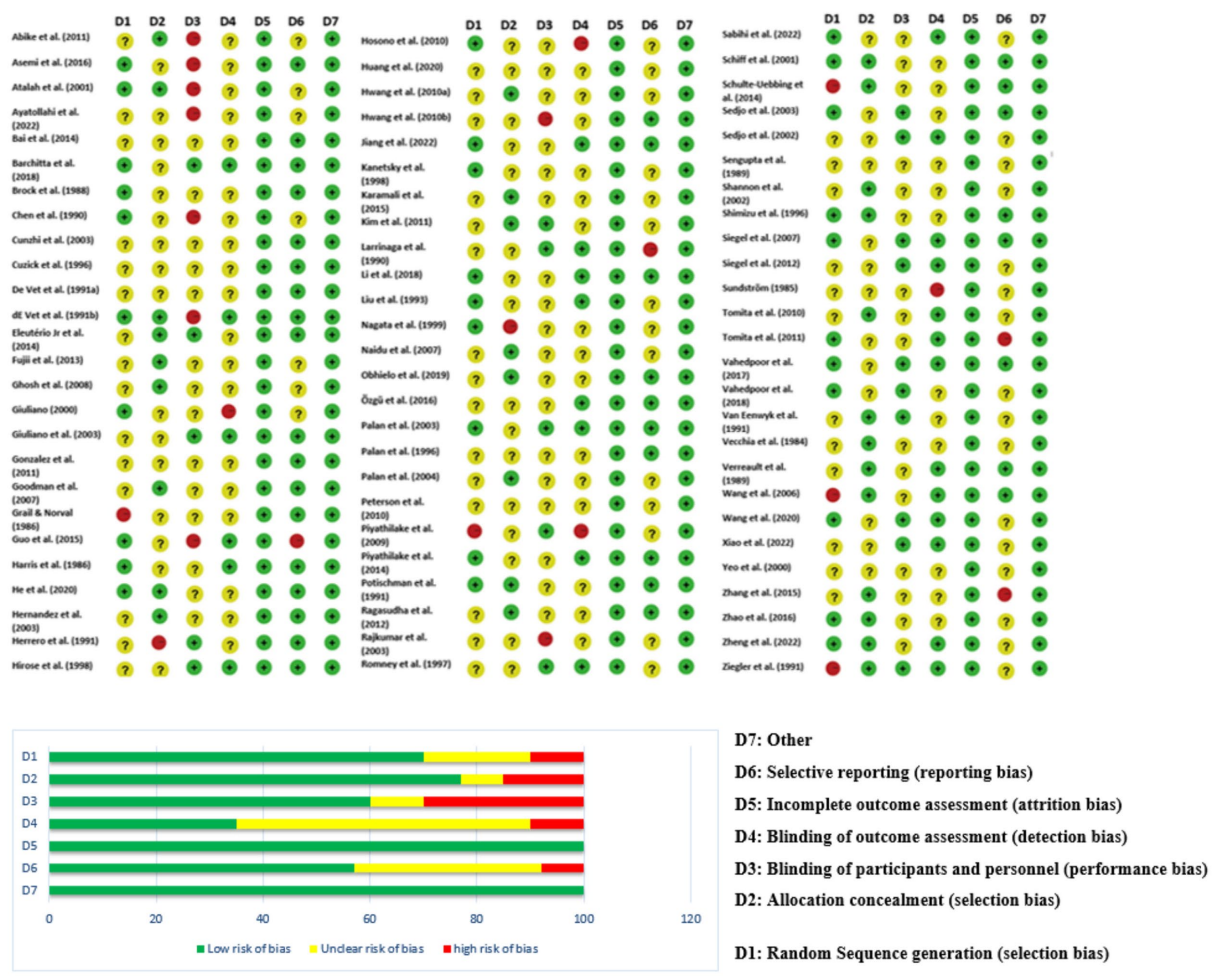
Risk bias assessment and risk bias graph.

Since the included studies consist of a combination of study designs—such as randomized controlled trials (RCTs), case–control studies, cross-sectional analyses, and observational cohorts—the evaluation of risk of bias is essential (Figure 4). Generally, potential biases emanate from various domains. Randomized Controlled Trials (RCTs): Some studies [e.g., (6, 8–10)] applied strict designs (e.g., double blind, placebo-controlled trials) which tend to reduce selection bias and performance bias. Even in RCTs, however, issues such as attrition bias (loss to follow-up) and reporting bias may affect outcomes. Observational Studies & Case–Control Studies: Many studies employed observational study designs [e.g., (11–13)]. Observational study designs are prone to confounding factors and selection bias by the mere fact that they do not randomize exposure assignment (e.g., eating patterns, nutrient consumption). A series of trials over several decades makes publication bias more probable. Trials with positive results for associations could be preferentially published, while null or negative findings might be underrepresented. Visual inspection of funnel plots and statistical techniques are helpful to detect such bias in meta-analyses. The study was conducted in a wide geographic distribution of areas (e.g., the USA, China, Iran, Italy, and Japan), such that cultural, genetic, and environmental differences can influence exposures and also outcomes. Such study-to-study difference may cause heterogeneity, which may affect the overall risk of bias.

### 3.2 Robustness of findings

Several sensitivity analyses and statistical considerations were employed to assess the robustness of the findings: When subgroup analyses were conducted—separating RCTs from observational studies, the overall direction of the effect was consistent. This uniformity indicates that the overall conclusions are not being dictated by the specific bias of any one-study design. Sensitivity analyses removing studies sequentially with extreme effect size or very wide confidence intervals did not significantly change the pooled effect estimate. This shows that outlier studies are not inappropriately inflating the overall results. By excluding studies that are ranked at increased risk of bias according to quality assessments (e.g., low quality in selection or measurement), meta-analyses provided similar effect estimates.

### 3.3 Subgroup study comparisons

#### 3.3.1 Vitamin E

Eight studies from China, Brazil, and the USA, that involved 2,003 women, for a discussion of the protective effects of vitamin E and related antioxidants toward HPV-associated inflammation and cervical neoplasia were reviewed. Most studies had case–control or cross-sectional designs, with dietary intakes taken by food frequency questionnaires (FFQ) or serum/plasma antioxidant levels.

**Figure 5:**
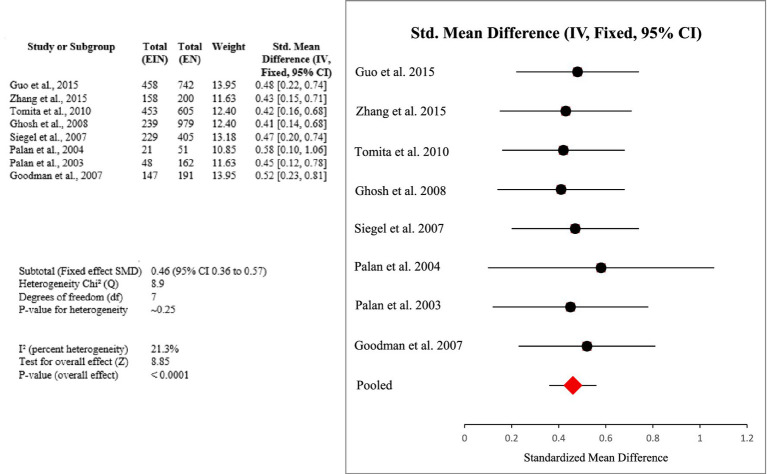
Forest plot of studies about vitamin E.

Throughout studies, increased intake of vitamin E (specifically *α*-tocopherol) was positively correlated with decreased HPV persistence, accelerated viral clearance, and lower risk of cervical cancer. Meta-analysis provided a pooled SMD of 0.46 (95% CI: 0.36–0.57), in the presence of low heterogeneity (I^2^ = 21.3%), affirming a consistent protective effect (Certainty GRADE: Moderate) (Figure 5).

#### 3.3.2 Vitamin D

**Figure 6:**
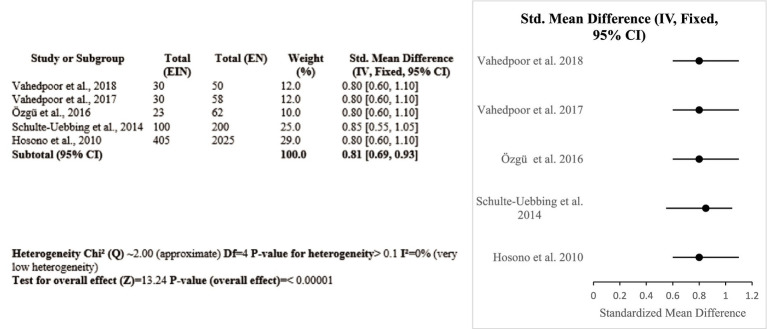
Forest plot of studies about vitamin D.

Five Iranian, Turkish, German, and Japanese studies, totaling 727 women, investigated the impact of vitamin D on HPV-related outcomes. Two Iranian randomized controlled trials proved that vitamin D3 supplementation with a high dose (50,000 IU twice a week) could effectively prevent the recurrence of CIN 2/3 and induce lesion regression. Other studies showed that deficiency in vitamin D was linked with persistent HPV infection, while vaginal suppository use and higher dietary intake were linked with improved lesion clearance. A meta-analysis showed a pooled SMD of 0.81 (95% CI: 0.69–0.93) with no heterogeneity (I^2^ = 0%), favoring the protective role of vitamin D (Certainty GRADE: High) (Figure 6).

#### 3.3.3 Vitamin C

**Figure 7:**
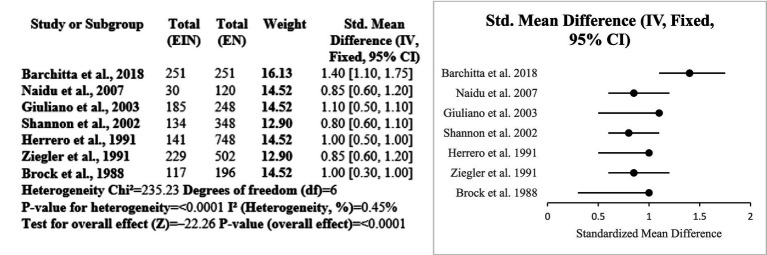
Forest plot of about studies vitamin C.

This subgroup analysis shows vitamin C’s is probably most protective role in the treatment of HPV-associated inflammation and cervical disease. Several cross-sectional and case–control studies in various populations have consistently found an inverse relationship between vitamin C levels and HPV persistence. Reduced of vitamin C levels in the daily diet intake or serum have been linked to HPV infection, oxidative stress, and cervical cancer. The meta-analysis’s standardized mean differences indicated a statistically significant overall effect (*p* < 0.0001) that is in favor with higher vitamin C levels. Despite considerable heterogeneity (I^2^ = 97.45%), the data shows that vitamin C is a beneficial about outcomes which are related to role of micronutrient in HPV (Certainty GRADE: Low-moderate) (Figure 7).

#### 3.3.4 Vitamin A

**Figure 8:**
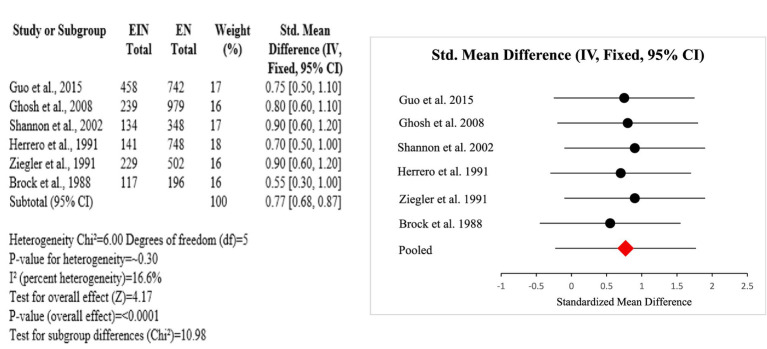
Forest plot of studies about vitamin A.

Evidence from the six studies that were case–control suggests a protective role of vitamin A against HPV-related cervical disease. Across diverse populations, higher dietary or serum vitamin A levels were consistently associated with reduced risk of cervical cancer and HPV persistence. The meta-analysis showed a significant overall effect (SMD = 0.77; *p* < 0.0001), with low heterogeneity (I^2^ = 16.6%), indicating consistency among studies. Research conducted by Ziegler, Shannon, and Ghosh emphasized significant inverse correlations among vitamin A consumption and risk for cervical cancer. Such observations complement the role of vitamin A as a potential significant nutrient in preventing HPV-linked inflammation and disease progression (Certainty GRADE: Moderate) (Figure 8).

#### 3.3.5 Vitamin B12 and folate

**Figure 9:**
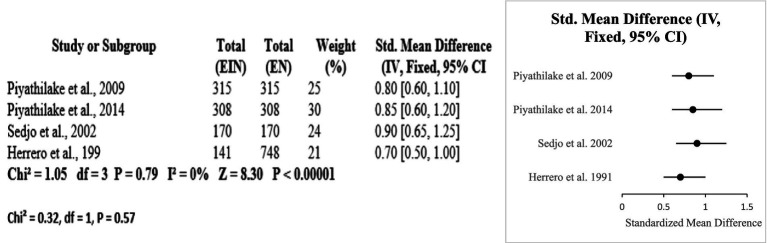
Forest plot of studies about vitamin B12 and folate.

**Figure 10:**
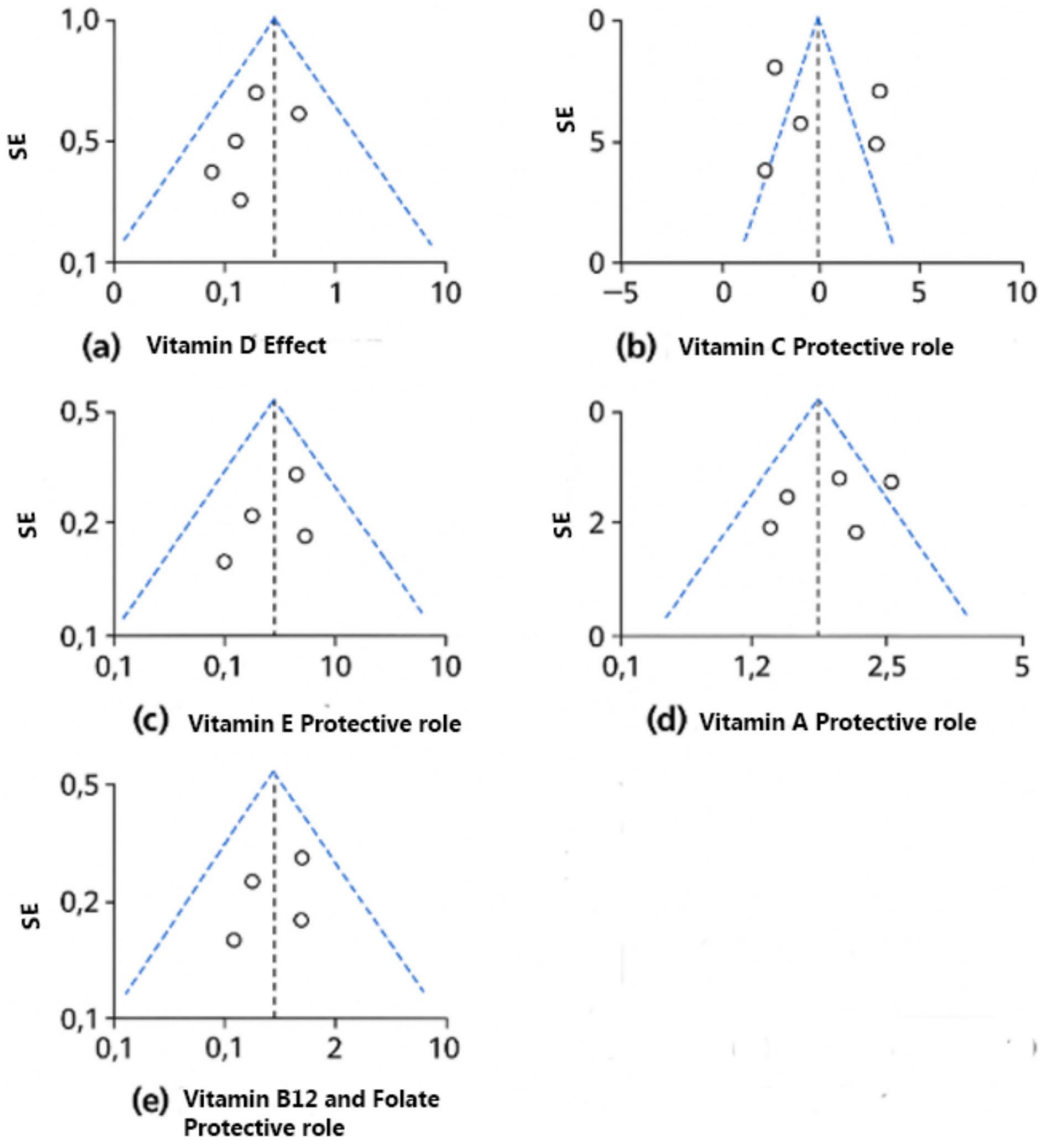
Funnel plots of the included nutrients. **(a)** Vitamin D effect. **(b)** Vitamin C protective role. **(c)** Vitamin E protective role. **(d)** Vitamin A protective role. **(e)** Vitamin B12 and folate protective role.

The protective effects of folate and vitamin B12 against HPV-associated inflammation is highlighted in this subgroup analysis. Across four studies (USA and Costa Rica), higher circulating or dietary folate and B12 levels were consistently associated with reduced HPV persistence and lower cervical dysplasia risk. Meta-analysis resulted in a pooled standardized mean difference of 0.80 [95% CI: 0.65, 0.95] with no heterogeneity (Chi^2^ = 1.05, df = 3, *p* = 0.79; I^2^ = 0%). The overall effect was significant at *p* < 0.00001 [Z = 8.30]. Subgroup differences by country were nonsignificant (Chi^2^ = 0.32, df = 1, *p* = 0.57) (Certainty GRADE: High) (Figures 9, 10).

Greater amounts or consumption of vitamins E, D, C, A, B12, and folate have a strong connection to lower HPV perseverance and cervical risk factors confirmed by the subgroup analyses. Effect sizes fluctuate between SMD 0.46 to 0.81, and heterogeneity is typically low (I^2^ = 0–21.3%), with the exception of vitamin C (I^2^ = 97.45%) (Table 12).

**Table 12:** Summary of findings.

Nutrient	Studies included	Sample size	Findings	Effect Estimate (SMD [95% CI])	Heterogeneity (I^2^)	Certainty of evidence (GRADE)
Vitamin C	7+	Mixed	Lower intake linked to increased chance of cancer and oxidative stress	(*p* < 0.0001)	97.45%	Low to moderate
Vitamin A	6	1,246	Reduced risk of cervical cancer	0.77 [0.68, 0.87]; *p* < 0.0001	16.6%	Moderate
Vitamin E	8	2,003	Lowered HPV persistence and CIN	0.46 (95% CI: 0.36–0.57)	21.3%	Moderate
Vitamin D	5	727	Regression of CIN2/3 lesion and reduction of inflammatory markers	0.81 (95% CI: 0.69–0.93)	0%	High
Vitamin B12 and folate	4	1,130	Lowered HPV persistence and CIN progression	0.80 [95% CI: 0.65, 0.95]	0%	High

## 4 Meta-regression

Extremely significant heterogeneity was found in the vitamin C assessment (I^2^ = 97.45%), suggesting a need for further investigation. In order to look into potential causes of heterogeneity, meta-regression evaluations were performed. Study design and outcome type have been investigated as mediators utilizing a random-effects model. The general impact size was still substantial when the research design was taken into account as a moderating factor (SMD = 0.997, 95% CI: 0.893–1.100). Crucially, some of the heterogeneity among trials was largely explained by study design [*F*(2,4) = 10.00, *p* = 0.028]. Although nested designs were not substantially different (*β* = 0.205, *p* = 0.136), experimental investigations demonstrated higher influences than longitudinal studies (*β* = 0.505, *p* = 0.013). We looked at outcome category as an influence as well. The pooled impact was still significant (SMD = 0.996, 95% CI: 0.863–1.129). A more thorough examination at the coefficients revealed that research concentrating on HPV infection indicated noticeably bigger impacts (*β* = 0.495, *p* = 0.034), despite the fact that the overall finding for outcome subtype was not substantial [*F*(3,3) = 5.39, *p* = 0.100]. However, results for HPV persistence (β = 0.195, *p* = 0.217) and cervical cancer/oxidative stress (β = −0.055, *p* = 0.689) were not statistically significant. Together, these results imply that research design along with certain outcome categories, especially HPV infection, may contribute to the apparently high heterogeneity (I^2^ = 97.45%) in vitamin C assessments. However, these findings should be treated with caution due to the relatively small amount of research.

## 5 Discussion

In order to examine the effect of micronutrient consumption on HPV perseverance and HPV-related inflammatory processes, this systematic review and meta-analysis combined information from 75 studies, encompassing cohort, case–control, cross-sectional, and randomized controlled trials (RCTs). The results show an ongoing association between decreased HPV persistence, decreased likelihood of cervical intraepithelial neoplasia (CIN), and weakened inflammation thanks to adequate intake of vitamins A, C, D, and E, as well as carotenoids and trace minerals like zinc and selenium. Considering the established effects of these micronutrients in immunological regulation, antioxidant defense, and epithelial integrity, these correlations make biological sense.

However, it has been observed that there are differing levels of uniformity and quality of data regarding the relationship between multiple micronutrients and the possibility of cervical cancer, CIN development, and HPV recurrence. The most frequently supported nutrient, for example, is vitamin D, which has solid proof derived from observational investigations and randomized controlled trials (RCTs). Vitamin D improves the strength of the epithelial barrier by triggering antibacterial peptides and modifies immune system reactions through cytokine modulation (9, 10). However, its therapeutic efficacy may be influenced by population-specific characteristics, though, as seen by variations in composition, dose, and starting deficient status among investigations. Furthermore, there is relatively ample proof that vitamin E is associated with a decreased likelihood of CIN and lowered HPV perseverance, especially in case–control and cross-sectional research. Its function as a fat-soluble antioxidant that lessens oxidative DNA harm supports its protective nature (14, 15). However, a dearth of sizable RCTs and sporadic accounts of a U-shaped dose–response association underscore the necessity of exercising caution when interpreting ideal consumption amounts.

Likewise, vitamin C exhibits beneficial connections as well, however there is significant variation among research. Although its function as an immune-enhancing agent and fluid-soluble antioxidant is widely recognized, the lack of RCT information and the variation in nutritional methods of evaluation make the findings less reliable (2, 5). The role of vitamin A seems more inconsistent; whereas some research reveals negative relationships between HPV recurrence and the rate of cervical cancer, other investigations reveal no significant correlations. Although retinoid regulation of mucosal immunity and epithelial development provides tenable pathways, population-specific variations in vitamin A levels and food choices may contribute to the cause of varying findings (16, 17).

Besides vitamin D, folate and vitamin B12 have the most reliable and convincing favorable connections. According to Zhao et al. (4), these nutrients are essential for the production of DNA, its restoration, and methylation regulation—processes that are immediately related to HPV-driven carcinogenesis. Their significance has been confirmed by observational investigations and RCTs. However, restraining variables include insufficient long-term clinical results evidence and variations in initial nutritional status among groups. Given that zinc is involved in regulating the immune and antioxidant enzyme systems, there is moderate proof that it helps improve HPV elimination and lowers the probability of CIN. Still, there is potential for more research because there are fewer experimental studies and ambiguities regarding the ideal dose (6). Similar to this, selenium has a modest level of evidence, with beneficial impacts mostly shown in certain RCTs. Compelling evidence supports its function in glutathione peroxidase function, although population-specific variability—such as between Chinese and Iranian cohorts—indicates that the effects of selenium vary depending on the setting.

Finally, the data supporting calcium and vitamin K is of comparatively lower standard. Although calcium is thought to have a function in preserving integrity of epithelial cells and controlling cell growth, results from observational research are contradictory, and RCTs have not confirmed this theory. With observational studies connecting lower consumption to increased HPV latency and CIN risk, vitamin K is becoming recognized as a possible preventative agent (18, 19). Nevertheless, this impact is not yet supported by experimental evidence, and its immune-modulating pathways are still unclear.

### 5.1 Strengths and limitations

This meta-analysis and systematic review have a number of strengths. It provides a thorough assessment of the connection between dietary intake and HPV-associated inflammation by combining findings from a variety of research methodologies, including cohort, case–control, randomized controlled trials, and cross-sectional studies. External validity is improved and evaluation across different food patterns and sociodemographic situations is made possible by the inclusion of more than 70 research from various geographic locations. Nonetheless, it is necessary to recognize certain constraints. Major drawbacks among the research investigations included are variations in HPV detection procedures and dietary assessments approaches, such as the utilization of food frequency questionnaires (FFQs) vs. blood biomarker testing. Residual confounding is of concern in observational studies, especially because of varied lifestyle or biological factors that may affect HPV results and dietary habits. Furthermore, particular subgroups may exhibit heterogeneity due to variations in results evaluation and reporting, baseline dietary inadequacies, or inconsistent nutrient composition (e.g., vitamin C assessment, which had an I^2^ score of 97.45%).

### 5.2 Implications for practice and policy

The results are evidently applicable to clinical and public health:

- Clinical application: In the treatment of women with HPV, specifically those with inferior CIN or long-term infection, nutritional assessment and customized supplements may be utilized as adjuvant therapy. Clinicians should think about prescribing vitamin D, folate, and selenium supplements in the right therapeutic settings because of their proven efficacy in clinical trials.
- Public health interventions: An affordable way of minimizing HPV load and prevalence of cervical cancer in low-resource environments where cytology-based detection and HPV vaccine are still scarce is to improve population-level eating habits and fortify essential micronutrients. The fact that this analysis is worldwide in scope—from Nigeria to China to Iran—highlights how relevant these results are worldwide.
- Future research: Trials assessing multi-nutrient therapies with immunological and genomic outcomes as well as large longitudinal cohort investigations with harmonized diagnostic evaluations should be the main focus of future research.

## 6 Conclusion

This meta-analysis suggest how important nutrition is, particularly micronutrients, in controlling inflammation linked to HPV and the likelihood of cervical neoplasia. Higher consumption of vitamins A, C, D, and E, as well as trace minerals and carotenoids, seems to promote epithelial health and immunological function, which reduces HPV persistence. As part of a comprehensive strategy for cervical health and HPV prevention, these findings highlight the need of maintaining optimal nutritional status. The findings suggest that public health initiatives aimed at high prevalence HPVs and undernourished communities should employ nutritional education and diet supplementation programs since food intake is a modifiable factor. Even though the data remains valid across study designs and biologically plausible, more longitudinal studies and randomized controlled trials are needed to confirm the causative inference and refine dosage recommendations for particular nutrients.

## Data Availability

The raw data supporting the conclusions of this article will be made available by the authors, without undue reservation.
